# A tourniquet-less technique using saline epinephrine irrigation system in an arthroscopic ACL reconstruction in patient with history of popliteal artery ligation

**DOI:** 10.1016/j.ijscr.2018.10.040

**Published:** 2018-11-01

**Authors:** Irissandya D. Atisuksma, Sholahuddin Rhatomy, Punto Dewo

**Affiliations:** a Resident of Orthopaedic and Traumatology, Faculty of Medicine, Universitas Gadjah Mada, Indonesia; b Department of Orthopaedic and Traumatology, Soeradji Tirtonegoro General Hospital Klaten, Indonesia; c Department of Orthopaedic and Traumatology, Faculty of Medicine, Universitas Gadjah Mada – Sardjito General Hospital Yogyakarta, Indonesia

**Keywords:** ACL reconstruction, Popliteal artery ligation, Tourniquet-less technique, Arthroscopic

## Abstract

**Introduction:**

Pneumatic tourniquets use in surgery to create a bloodless surgical field. The majority of orthopaedic surgeon use a tourniquet inflated above systemic blood pressure during arthroscopic anterior cruciate ligament (ACL) reconstruction. A tourniquet use is not free of complications. The disadvantage of tourniquet application include an increased risk of vascular injuries. An arthroscopic ACL reconstruction in patient with history of popliteal artery ligation is challenging. The popliteal artery is the major contributor to the blood supply of the knee joint. This arthroscopic ACL reconstruction does not use tourniquets to preserve the collateral circulation that provides blood supply to the leg and prevent the vascular injuries in patient with history of popliteal artery ligation.

**Presentation of case:**

A-23-year-old female brought to the General Hospital with ACL rupture caused by car accident. The patient had a history of knee dislocation with an open wound and rupture of popliteal artery. The patient underwent open reduction surgery of her right knee joint by orthopaedic surgeon and popliteal artery repair by vascular surgeon. The reduction of the knee joint went good, but the popliteal artery repair was failed and the patient underwent popliteal artery ligation 1 week later. The patient received non-operative treatment for her ACL rupture. After 2 years, she did not get a good improvement to her knee and the patient still felt a giving way sensation and unstability of her knee. Then the patient transferred to the General Hospital for ACL reconstruction, but the surgery required a special consideration in the technique of ACL reconstruction because of the history of popliteal artery ligation.

**Discussion:**

Positioning of the patient for ACL reconstruction The patient supine on an operating table. The leg to undergo surgery has no tourniquet placed because the patient had no popliteal artery and this operation needs to preserve and prevent the vascular ischemia of the collateral artery. Operating room set up with the patient prepped and draped for the diagnostic arthroscopy. It shows a normal cartilage, rupture of the ACL and PCL, rupture of body of the lateral meniscus in the white zone and rupture of body of the medial meniscus in the white zone. To make the bloodless arthroscopic field, cold saline and epinephrine pumped into the knee. Partial meniscectomy of the lateral and medial meniscus was performed. Single bundled ACL reconstruction was performed using hamstring autograft of the contralatelal site with the diameter was 8 mm and fixated by XO button and bioscrew (ConMed). After six months follow up, the patient did not feel giving way, catched, or locking. The patient had a good vascularity of the right lower extremity. There is improvement in Modified Cincinnati Rating System. The pre operative score was 49. The post operative score was 90. Tegner Lysholm Knee Scoring system before surgery was 35 and post operative score was 91. IKDC Scoring before surgery was 21,8 and the score had a good improvement. The IKDC Scoring after surgery was 73,6.

**Conclusion:**

ACL reconstruction in patient with popliteal artery ligation is challenging. A tourniquet-less technique using a cold saline and epinephrine solution can be successfully done for pressure controlled into the knee to preserve the collateral arteries flow to the distal limb while still permitting acceptable visual in operative field with good outcome after the surgery.

## Highlights

• ACL injuries are the most common devastating knee ligament injuries among sports persons.
• The ideal successful ACL reconstruction is expected to provide a stable pain-free knee and the patient being able to return to full function.
• ACL reconstruction in patient with popliteal artery ligation is challenging.
• A tourniquet-less technique using saline epinephrine irrigation system in arthroscopic ACL reconstruction in patient with history of popliteal artery ligation.

## Graphical abstract

**Figure:**
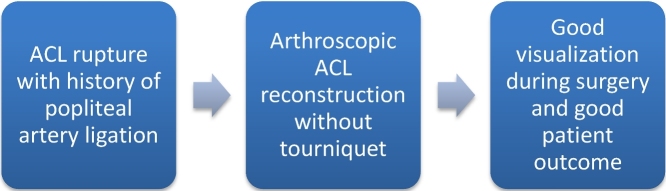

## 1 Introduction

### 1.1 Background

Noordin et al. reported the history of tourniquet use in surgery, they mentioned Joseph Lister to be the first to use a tourniquet to create a bloodless surgical field in 1864 with the advent of general anesthesia. Harvey Cushing introduced the first inflatable (pneumatic) tourniquet, thereby permitting tourniquet pressure to be monitored and manually controlled. Since then, Pneumatic tourniquets have been used for elective surgery to reduce intra-operative blood loss and improve visibility in the surgical field. The majority of orthopedic surgeons use a tourniquet inflated above systemic blood pressure during arthroscopic anterior cruciate ligament (ACL) reconstruction.

However tourniquet use is not free of complications. McEwen 2014, reported that the disadvantages of tourniquet application include an increased risk of nerve palsy, vascular injury, muscle damage, postoperative swelling and stiffness. Ols-zewski et al. investigated irrigation with a dilute solution of epinephrine in saline solution (1 mg/L) delivered by gravity flow during routine knee arthroscopy. A satisfactory bloodless field was reported in thirty of thirty-seven patients treated with an intra-articular anesthetic and epinephrine. This case study is explored to try and understand the technique of arthroscopic anterior cruciate ligament reconstruction in patient with history of popliteal artery ligation.

### 1.2 Problem

To suggest solutions technique of arhroscopic ACL reconstruction in patient with popliteal artery ligation

To evaluate how the tourniquet-less technique in arthroscopic ACL reconstruction help to increase surgeon’s visual field. This work has been reported in line with SCARE criteria [1].

## 2 Literature review

### 2.1 Definition

The ACL is an important component for normal kinematics of the knee joint. The primary function of the ACL is to restrain anterior translation of the tibia on the femur in open chain activities and perhaps more importantly, restrain posterior translation of the femur when the tibia is fixed, i.e. closed chain activity [2]. The mechanism of injury for an ACL tear is usually associated with a deceleration or a change of direction on the planted lower extremity i.e. pivoting [2,3].

### 2.2 Anatomy and physiology

The stability of the knee is enhanced by a complex arrangement of ligaments. The cruciate ligaments are crucial to anteroposterior stability, and the collateral ligaments provide varus/valgus stability. Each cruciate ligament is made up of two portions, or bundles. The anterior bundles of the ACL and posterior cruciate ligament (PCL) are tight in flexion. The PCL has an anterolateral bundle and the ACL an anteromedial bundle. Thus, the ACL is composed of an anteromedial portion that is tight in flexion and a posterolateral portion that is tight in extension. The PCL has an anterolateral portion that is tight in flexion and a posteromedial portion that is tight in extension. The PCL lies between the ligament of Humphrey (anterior) and the Wrisberg ligament (posterior). The posterolateral corner (PLC) comprises the arcuate ligament, popliteus, posterolateral capsule, lateral collateral ligament (LCL), popliteofibular ligament, and lateral head of the gastrocnemius. Injuries to the PCL and PLC provide key testable material. Isolated injuries to the PCL cause the greatest instability at 90° of knee flexion. Combined PCL and PLC injuries result in increasing instability as the knee is flexed from 30 to 90°. Isolated PLC injuries result in increasing instability that is most notable at 30°, with instability decreasing as the knee is flexed to 90°.

**Fig. 1:**
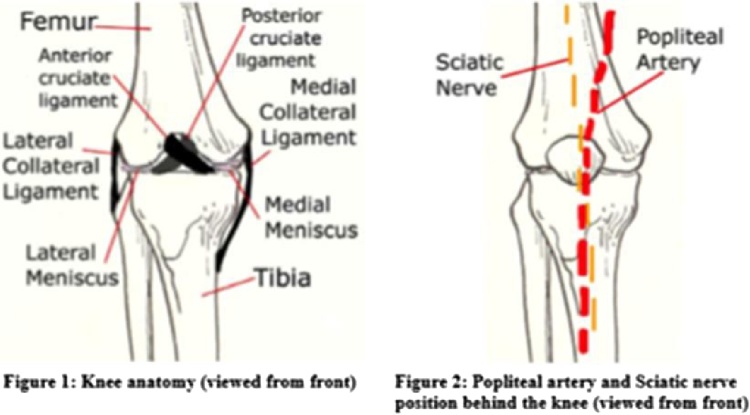
Knee anatomy.

The popliteal artery, sciatic nerve and branches pass very closely at the back of the knee joint and are at risk of injury if the knee dislocates (usually with rupture of the major stabilisers) [4] (Fig. 1).

### 2.3 ACL injury

ACL injuries are, probably, the most common devastating knee ligament injuries among sports persons, with a reported 90,000 ACL reconstructions performed annually, in the United States alone. Usually these injuries are isolated, mainly in non- contact sports, but may often be a part of more complex ligamentous injuries. They occur more often in contact sports, such as football, and road traffic accidents. These injuries are most likely to lead to the need for surgery and are therefore discussed in more detail here.

Injury usually occurs in athletic activities with both a contact and non- contact mode of trauma. Football, basketball and skiing are sports where ACL ruptures are common. There is often a history of a sudden deceleration injury with a “pop” and the knee usually swells up fairly quickly over an hour or so, with a tense painful haemarthrosis of the joint. Usually the patient is unable to continue playing following this injury.

Late symptoms include the feeling of sudden giving way of the knee, which may occur on any sudden twisting or turning movement. Intermittent symptoms are also associated with mild recurrent swelling, with giving way, and may be entirely pain free. If an ACL injury is associated with a concomitant meniscal tear or complex capsular disruption the symptoms may reflect that injury, with a locked knee or localised joint line pain [4].

### 2.4 Etiology

Ligament and meniscal injuries are usually of traumatic onset (sporting injuries or road traffic accidents). A history of a sporting trauma is usual; otherwise, the traumatic event may be a direct injury to the front of the knee in a dashboard injury or motorcycle accident. The trauma may be direct, as in rugby tackles, or indirect, such as twisting injuries in falls while skiing, with no other person involved.

Injuries may occur with a single ligament involvement or quite often involve multiple ligaments. Complex injuries are more common with severe high velocity trauma, for example, in road traffic accidents, motorcycle.

### 2.5 ACL reconstruction

The ACL is the most commonly reconstructed ligament of the knee [3]. It has been estimated that more than 100,000 new ACL injuries occur each year [3]. An injury to the ACL can result in significant functional impairment [5]. Although reconstruction of the acutely torn ACL (<3 weeks after injury) has fallen out of favor [6], failure to reconstruct the ligament at all can lead to recurrent bouts of instability, damage to the meniscus and articular cartilage, and may accelerate. The progression of osteoarthritis for the active individual [7,8,5]. Diagnostic tests used to confirm trauma to the ACL include the Lachman test [3,[8], [9], [10]], the prone Lachman test [11], the pivot shift test [3,[8], [9], [10]], and the KT1000/2000 arthrometer [3,8,9]. Magnetic Resonance Imaging (MRI) is also used because it provides the fine soft tissue detail necessary for a definitive diagnosis [10]. Once damage to the ACL has been confirmed, indications for the reconstruction of the ACL include [3,10]:

• The high performance athlete
• The young/healthy active individual
• The individual involved in sports that require pivoting and jumping
• The individual involved in recreational activities >5 h/week
• The individual with 3 or more episodes of instability per year
• The individual with an arthrometer assessment of 5 mm more displacement than the uninvolved knee
• The individual that failed a conservative rehabilitation program. In contrast, the predictors of a less than optimal surgical outcome may include [10]:
• Sedentary lifestyle
• Obesity
• Open growth plates
• Degenerative joint disease
• Coexisting medial meniscus tear
• Failure to comply with pre-operative rehabilitation.
  Surgeons employ numerous techniques for reconstruction of the ACL [12].

### 2.6 Prognosis

A 5–10 year follow-up of ACL deficient knees showed that on average, 33% of patients had pain and “giving way” with activities of daily living (ADL), 33% had symptoms mainly on sporting activities and 33% had no symptoms in either sports or in ADL.

Sixty percent of ACL injured patients have an associated meniscal tear, 20% have other ligamentous injuries and 10–20% have associated osteochondral fracture [13]. Late development of degenerative osteoarthritis of the knee is more common after ACL injuries. The factors that increase the risk of developing arthrosis include the following:

• Number of hours involved in jumping, pivoting, hard cutting and lateral motion pre-injury
• Associated meniscal or osteochondral injury
• Patients refusing to consider activity modification
• More frequent incidence of the knee “giving way”
• Heavy manual work involving climbing, walking on uneven surfaces etc. post injury

An unreconstructed ACL injury, which remains symptomatically unstable, will have a greater incidence of meniscal tears and osteochondral injury due to the “secondary trauma”.

No study has shown that ACL reconstruction protects against articular cartilage degeneration and secondary osteoarthritis. However, the incidence of secondary injury is reduced by a successful reconstruction and the return to previous level of sports is high amongst well- motivated athletes [14].

The ideal successful ACL reconstruction is expected to provide a stable pain-free knee and the patient being able to return to full function, including sports at the pre-injury level. It should also prevent the subsequent development of secondary injury and arthritis. Most studies in the literature generally report a 90–95% success rate [15].

## 3 Material and methods

### 3.1 Case

**Fig. 2:**
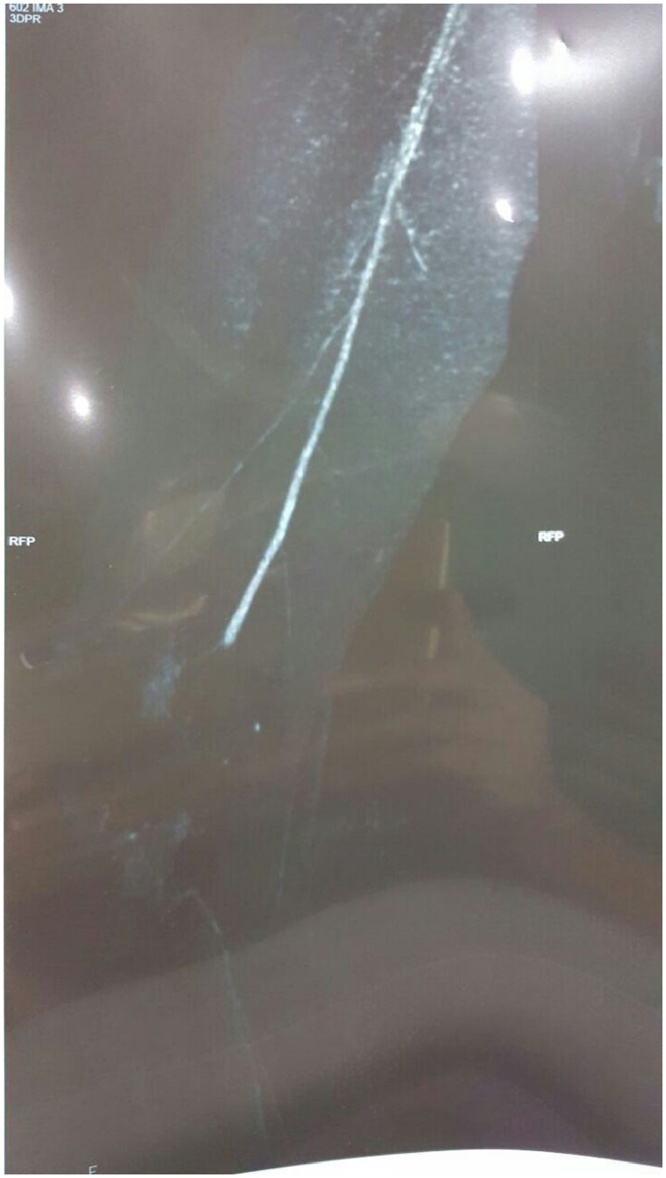
Angiography of the patient that showed the popliteal artery was ligated.

A-23-year-old female brought to our hospital with ACL rupture caused by car accident. The patient had a history of knee dislocation with an open wound and rupture of popliteal artery. The patient underwent open reduction surgery of her right knee joint by orthopaedic surgeon and popliteal artery repair by vascular surgeon. The reduction of the knee joint went good, but the popliteal artery repair was failed because there was leakage, the distal popliteal artery was damage and the angiography (Fig. 2) showed the blockage below the popliteal artery. Data showed that limb revascularizations in both military and civilian populations were mostly done by autogenous bypass. Popliteal artery injury with early identification of limbs at risk, shown to be beneficial to do: early four compartment fasciotomy, temporary intra-luminal shunting, definitive repair of concomitant venous injuries [16], because there was good distal vascularity from the collateral artery of the patient and there was no sign of ischemia with the popliteal artery blockage then the patient underwent popliteal artery ligation 1 week later.

The patient received non-operative treatment for her ACL rupture. After 2 years, she did not get a good improvement to her knee and the patient still felt a giving way sensation and unstability of her knee. Then the patient transferred to our hospital for ACL reconstruction, but the surgery required a special consideration in the technique of ACL reconstruction because of the history of popliteal artery ligation (Fig. 2).

### 3.2 Methods

**Fig. 3:**
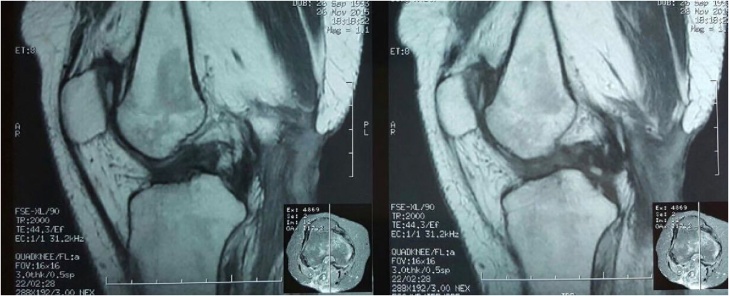
MRI T2-weighted.

**Fig. 4:**
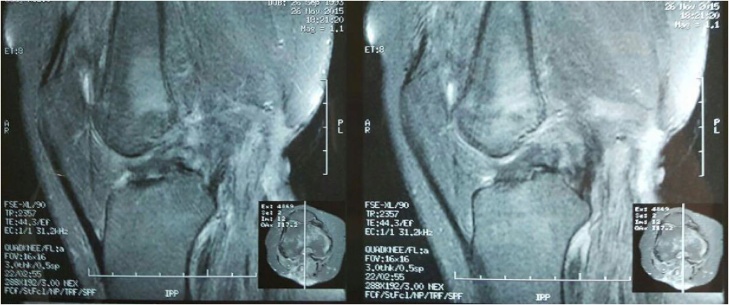
MRI T1-weighted.

Written informed consent was obtained from the patient for publication of this case report and accompanying images. A copy of the written consent is available for review by the Editor-in-Chief of this journal on request. Patient had diagnosis confirmed before operation by clinical evidence alone. MRI was performed in this patients where concomitant injuries were suspected (Fig. 3, Fig. 4).

At surgery patient had spinal anesthesia with the spinal intravenous spinal anesthesia agents. Local anesthesia did not performed in this procedure because ACL reconstruction can be performed no matter the type of anesthesia although some research recommend the use of dexamethasone during the induction phase periarticularly [17].

**Chart 1:**
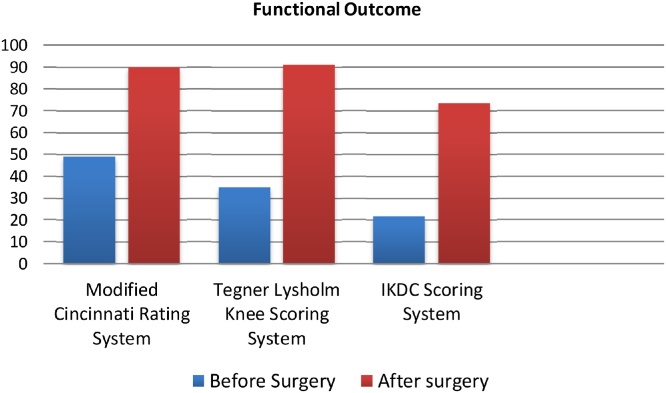
Functional score before and after surgery.

Single dose, prophylactic, intravenous antibiotics were administered intra­operatively. Positioning of the patient for ACL reconstruction. The patient supine on an operating table. The leg to undergo surgery has no tourniquet placed because the patient had no popliteal artery and this operation needs to preserve and prevent the vascular ischemia of the collateral artery. Operating room set up with the patient prepped and draped for the diagnostic arthroscopy. It shows a normal cartilage, rupture of the ACL and PCL, rupture of body of the lateral meniscus in the white zone and rupture of body of the medial meniscus in the white zone. To make the bloodless arthroscopic field, cold saline and epinephrine pumped into the knee. Partial meniscectomy of the lateral and medial meniscus was performed. Single bundled ACL reconstruction was performed using hamstring autograft of the contralatelal site with the diameter was 8 mm and fixated by XO button and bioscrew (ConMed) (Table 1, Chart 1).

**Table 1:** Physical examination before and after surgery.

Physical Examination	Before Surgery	After surgery
Drawer Test	+++	+
Lelli Test	+	−
Lachman Test	+++	−
Dial Test	−	−

## 4 Discussion

After six months follow up, the patient did not feel giving way, catched, or locking. The patient had a good vascularity of the right lower extremity.

There is improvement in Modified Cincinnati Rating System. The pre operative score was 49. The post operative score was 90. Tegner Lysholm Knee Scoring system before surgery was 35 and post operative score was 91. IKDC Scoring before surgery was 21,8 and the score had a good improvement. The IKDC Scoring after surgery was 73,6. The patient had a good improvement after six months follow up.

ACL reconstruction using arthroscopy need a placement of tourniquet to minimize bleeding and provide bloodless arthroscopic field. Because the genicular arteries could not compensate for a ligated popliteal artery, the ACL reconstruction of this patient do not use a tourniquet.

During ACL reconstruction, cold sterile saline solution with ephinephrine is pumped into the knee through one incision to wash blood from the area and it works successfully to minimize bleeding the arthroscopic field and preserve the collateral arteries around the knee.

After the surgery the patient had rehabilitation therapy. It includes knee ROM exercise, strengthening exercise of the knee joint, and weight bearing exercise.

Six months after the surgery the patient has a good improvement in IKDC Subjective Knee Evaluation, Modified Cincinnati Rating System and Tegner Lysholm Knee Scoring Scale.

## 5 Conclusion

ACL reconstruction in patient with popliteal artery ligation is challenging. A tourniquet-less technique using a cold saline and epinephrine solution can be successfully done for pressure controlled into the knee to preserve the collateral arteries flow to the distal limb while still permitting acceptable visual in operative field with good outcome after the surgery.

## Conflicts of interest

I have nothing to declare in this category.

## Sources of funding

I would like to thank Anggun Esti Wardani, MD for his assistance in patient radiologic examination taking.

## Ethical approval

Ethical approval has been given by medical and health research ethics committee (MHREC) Faculty of Medicine Gadjah Mada University Dr. Sardjito General Hospital. The reference number is KE/FK/0531/EC/2017.

## Consent

Informed consent is available.

## Author contribution

Sholahuddin Rhatomy contributes in study concept and data collection.

Punto Dewo contributes in study concept.

Irissandya Dyah Atisuksma contributes in interpretation, writing the paper.

## Registration of research studies

Research registry 4342.

## Guarantor

Sholahuddin Rhatomy, MD.

## Provenance and peer review

Not commissioned, externally peer reviewed.

## References

[1] Agha R.A., Fowler A.J., Saetta A., Barai I., Rajmohan S., Orgill D.P., for the SCARE Group (2016). The SCARE statement: consensus-based surgical case report guidelines. Int. J. Surg..

[2] Hiemstra L.A., Webber S., MacDonald P.B. (2000). Knee strength deficits after hamstring tendon and patellar tendon anterior cruciate ligament reconstruction. Med. Sci. Sports Med..

[3] Bach B.R., Boonos C.L. (2001). Anterior cruciate ligament reconstruction. Assoc. Oper. Room Nurs. J..

[4] Chouduri (2008). Knee ligament damage. Synopsis of Causation.

[5] Lephart S.M., Kocher M.S., Harner C.D., Fu F.H. (1993). Quadriceps strength and functional capacity after anterior cruciate ligament reconstruction. Am. J. Sport Med..

[6] Ramsdell V.J., Tietjen R. (1994). Anterior cruciate ligament: past, present, and future. Curr. Concepts Sports Med..

[7] Brown C.H., Sklar J.H. (1998). Graft selection: nonpatellar alternatives gain popularity. Biomechanics.

[8] Corry I.S., Webb J.M., Clingeleffer A.J., Pinzewski L.A. (1999). Arthroscopic reconstruction of the anterior cruciate ligament: a comparison of patellar tendon autograft and four-strand hamstring tendon autograft. Am. J. Sports Med..

[9] Barrett G.R., Boojin F.K., Hartzog C.W., Nash C.R. (2002). Reconstruction of the anterior cruciate ligament in females: a comparison of hamstring versus patellar tendon autograft. Arthrosc. J. Arthrosc. Relat. Surg..

[10] Bartolozzi A. (1993). Rothman institute information packet (for patients). Anterior cruciate ligament injuries. Presented at Pennsylvania Hospital Conference on ACL Injuries.

[11] Norkus S.A., Swartz E.E., Floyd R.T. (2002). Advantages of the prone Lachman test. Athl. Ther. Today.

[12] Aune A.K., Holm I., Risberg M.A., Jensen H.K., Steen H. (2001). Four-strand hamstring tendon autograft compared with patella-tendon-bone autograft for anterior cruciate ligament reconstruction: a randomized study with 2-year follow-up. Am. J. Sports Med..

[13] Noyes F.R., Bassett R.W., Grood E.S. (1980). Arthroscopy in acute traumatic hemarthrosis of the knee. Incidence of anterior cruciate tears and other injuries. J. Bone Joint Surg. Am..

[14] Noyes F.R., Matthews D.S., Mooar P.A., Butler D.L. (1983). The symptomatic anterior cruciate-deficient knee. Part II: the results of rehabilitation, activity modification and counselling on functional disability. J. Bone Joint Surg. Am..

[15] Miller R.H., Canale S.T. (2003). Knee injuries. Campbell’s Operative Orthopaedics.

[16] Reis P.E.E.O. (2015). Surgical treatment of traumatic injury of the artery and popliteal vein - a case report. J. Clin. Case Rep..

[17] Baverel L., Cucurulo T. (2016). Anesthesia and analgesia methods for outpatient anterior cruciate ligament reconstruction. Orthop. Traumatol.: Surg. Res..

